# Mining Biosynthetic Gene Clusters in *Carnobacterium maltaromaticum* by Interference Competition Network and Genome Analysis

**DOI:** 10.3390/microorganisms10091794

**Published:** 2022-09-06

**Authors:** Marco Túlio Pardini Gontijo, Nancy E. Ramia, Alexis Dijamentiuk, Annelore Elfassy, Samir Taha, Cécile Mangavel, Anne-Marie Revol-Junelles, Frédéric Borges

**Affiliations:** 1 Université de Lorraine, LIBio, F-54000 Nancy, France; 2 Instituto de Biologia, Universidade Estadual de Campinas, 255, Rua Monteiro Lobato, Campinas 13083-862, Brazil; 3 Laboratoire de Biotechnologies Appliquées, EDST, Université Libanaise, Tripoli 1300, Lebanon

**Keywords:** biopreservation, bioprotection, bacteriocin, NRPS-PKS, lactic acid bacteria, network, genome mining

## Abstract

*Carnobacterium maltaromaticum* is a non-starter lactic acid bacterium (LAB) of interest in the dairy industry for biopreservation. This study investigated the interference competition network and the specialized metabolites biosynthetic gene clusters (BGCs) content in this LAB in order to explore the relationship between the antimicrobial properties and the genome content. Network analysis revealed that the potency of inhibition tended to increase when the inhibition spectrum broadened, but also that several strains exhibited a high potency and narrow spectrum of inhibition. The *C. maltaromaticum* strains with potent anti-*L. monocytogenes* were characterized by high potency and a wide intraspecific spectrum. Genome mining of 29 strains revealed the presence of 12 bacteriocin BGCs: four of class I and eight of class II, among which seven belong to class IIa and one to class IIc. Overall, eight bacteriocins and one nonribosomal peptide synthetase and polyketide synthase (NRPS-PKS) BGCs were newly described. The comparison of the antimicrobial properties resulting from the analysis of the network and the BGC genome content allowed us to delineate candidate BGCs responsible for anti-*L. monocytogenes* and anti-*C. maltaromaticum* activity. However, it also highlighted that genome analysis is not suitable in the current state of the databases for the prediction of genes involved in the antimicrobial activity of strains with a narrow anti-*C. maltaromaticum* activity.

## 1. Introduction

Lactic acid bacteria (LAB) are widely industrially used as bioprotective cultures due to their capacity to synthesize antimicrobial compounds, such as primary metabolites resulting from primary metabolism and bacteriocins [1]. Bacteriocins are ribosomally synthesized bacterial peptides or proteins produced by bacteria that may favor niche occupancy over competitors by (i) conquering or replacing a bacterial competitor in an ecological niche; or (ii) maintaining an already colonized niche [2,3]. Bacteriocins can be classified into three classes [4]: class I comprises peptides with less than 50 amino acids that are extensively post-translationally modified; class II refers to small (<10 kDa) and heat-stable, non-modified peptides; and class III brings together large (>10 kDa) and heat-labile proteins.

Although LAB are reported to produce mainly specialized metabolites of the bacteriocin type, the genome of several strains has been described to harbor nonribosomal peptide synthetase (NRPS) and polyketide synthase (PKS) biosynthesis gene clusters (BGCs) [5,6,7]. Nonribosomal peptides (NRPs) comprise a diverse range of natural products of bacterial or fungal origin [8]. Polyketides (PKs) form a large family of molecules synthesized by bacteria, fungi, and plants. Both NRPs and PKs exhibit a wide range of biological functions, including antimicrobial activity [9]. NRPS and PKS systems are typically composed of several enzymatic domains organized in subunits [10]. The basic unit incorporated in the polymers consists of amino acids for NRPs and acyl-coenzyme A for PKs [11].

*Carnobacterium maltaromaticum* is a non-starter LAB [12,13] of technological interest in cheese production as non-starter culture [14,15,16], as bioprotective cultures in Ricotta fresca cheese [16], meat [17] and seafood [18], or as probiotics in fish [19,20] and rabbits [21]. The biopreservative potential of the *C. maltaromaticum* species is partly due to the production of bacteriocins of wide inhibition spectra [16,18]. *Carnobacterium* spp. are reported to produce the class I Carnocin UI49 [22], carnolysin A1-A2 [23] and carnocyclin A [24]; the class IIa carnobacteriocins BM1 and B2 [25], piscicolin 126 [26], piscicocins CS526 [27], V1a and V1b [28]; and the class IIc piscicolin 61 [29] and carnobacteriocin A [30].

Several strains of *C. maltaromaticum* are commercially available for biopreservation purposes. The strain *C. maltaromaticum* CB1, which produces the three bacteriocins carnocyclin A, piscicolin 126, and carnobacteriocin BM1, is commercially used to inhibit spoilage and the pathogenic bacterium *Listeria monocytogenes* during the storage of vacuum packaged meat products [21,31]. Lately, the two strains of *C. maltaromaticum* F88 and B10 (CNCM I-5242 and CNCM I-5243, respectively) were selected for potent anti-*L. monocytogenes* in soft cheeses and one of these strains is commercially available for biopreservation applications [32]. In packed Ricotta fresca cheese, a commercial biopreservative Lyofast CNBAL containing *Carnobacterium* spp. showed a significant reduction of *Pseudomonas* spp. of 0.83 log without changing the intrinsic parameters and the physicochemical composition of the product [16].

Besides their interest in biopreservation, the impact of the production of inhibitory compounds between non-pathogenic food microorganisms has been studied lately [33]. High-throughput competition assays allowed for investigating 5329 pairwise interactions within a collection of 73 *C. maltaromaticum* strains that revealed that competition is major within this species. Approximately 56% of the tested strains showed antagonistic activity toward at least one other strain. A high diversity of the inhibitory and sensitivity spectra was found with a majority of narrow spectra, suggesting that interference competition plays a major role in this species. In addition, the network of interference competition in this species is not random but rather structured according to a nested pattern. However, the compounds involved in the inhibitory activities recorded in this study are not well known [33].

The aim of this study was to investigate the relationship between the antimicrobial properties described in a competition network and the genomic features of 29 *C. maltaromaticum* strains, in order to gain insight into the potential specialized antimicrobial metabolites produced by these strains.

## 2. Materials and Methods

### 2.1. Bacterial Strains and Culture Conditions

The strains of *C. maltaromaticum* (Table S1) and *L. monocytogenes* EGDe were cultivated in Trypticase Soy Broth medium (bioMérieux, Marcy-l’Etoile, France) supplemented with 6 g·L^−1^ of bacto-Yeast Extract (TSBYE) at 30 °C without agitation. The cultures in broth were either performed in standing tubes containing 10 or 50 mL of TSBYE medium or in 96-well sterile microplates filled with 195 µL of TSBYE per well. Cultures on a solid medium were performed in TSBYE supplemented with 15 g·L^−1^ agar and incubated at 30 °C in aerobic conditions.

### 2.2. Competition Assays and Data Processing

The competition assays and the data processing were performed as previously described [33]. When the strains of *C. maltaromaticum* were used as the target, the inhibition potency was expressed as the growth inhibition indicator (GII), which was calculated as t2-t1, where t2 is the time (in min) required for the receiver strain culture to reach an optical density value of 0.2 at 595 nm (OD_595 nm_) when it is cultivated in a fresh medium supplemented with cell-free supernatant, and t1 the time (in min) required for the receiver strain to reach an OD_595 nm_ value of 0.2 when cultivated in fresh medium only. As previously described, GII values higher than the threshold of 300 min were considered inhibitions [33]. When the strain *L. monocytogenes* EGDe was used as the target, the classification of *C. maltaromaticum* sender strains as inhibitory or non-inhibitory was performed by non-supervised clustering. To do so, the software R (version 4.1.0, sourced at CRAN) [34] and the package factoextra (version 1.0.7) [35] were used to define the optimal number of groups with the silhouette method and to perform kmeans clustering.

### 2.3. Genome Sequencing and Analysis

The genome of the *C. maltaromaticum* strains was sequenced using an Illumina MiSeq sequencer (Eurofins genomics, Les Ulis, France) in order to generate paired-end reads. De novo assembly was performed using QIAGEN CLC Genomics Workbench 10. Genome annotations were carried out using the platform MicroScope (Evry-Courcouronnes, France) [36,37,38,39].

### 2.4. Data Analysis

Secondary metabolite gene clusters were predicted using BAGEL [40,41,42,43] and antiSMASH [44,45,46,47,48]. Genome clustering was conducted on the MicroScope platform which uses an approach that originates from network science called community detection. The resulting pairwise inter-genomics distances, which are approximately equal to 1-ANI (Average Nucleotide Identity), are used to construct a tree using the neighbour-joining method [39]. The heatmaps were constructed with the R package ComplexHeatmap (version 2.12.1, sourced at Bioconductor) [49]. Hierarchical clustering of the inhibitory profiles was performed by using Euclidean distances and the average method with the R package ComplexHeatmap [49]. Simple linear regression modelling, descriptive statistics, ANOVA, and Tukey’s test were performed in base R [34]. The data manipulation and the graphical representations were performed using the R packages tidyverse (version 1.3.1, sourced at CRAN) [50], ggprism (version 1.0.3, sourced at CRAN) [51], ggrepel (version 0.9.1, sourced at CRAN) [52], and viridis (version 0.6.2, sourced at CRAN) [53].

### 2.5. Accession Numbers

The accession numbers of enterocin F4-9, carnobacteriocin BM1, carnobacteriocin B2, piscicolin 126, hiracin JM79, lactococcin 972, are BAR87969, AAA23014.1, AAB81310.1, AAX21354.1, Q0Z8B6.1, WP_000726502.1, respectively. The genome sequences and annotations are publicly available on the MicroScope MaGe web portal (Table S1, https://mage.genoscope.cns.fr, last accession on 21 July 2022).

## 3. Results

### 3.1. Adjacency Matrix of Inhibition

**Figure 1:**
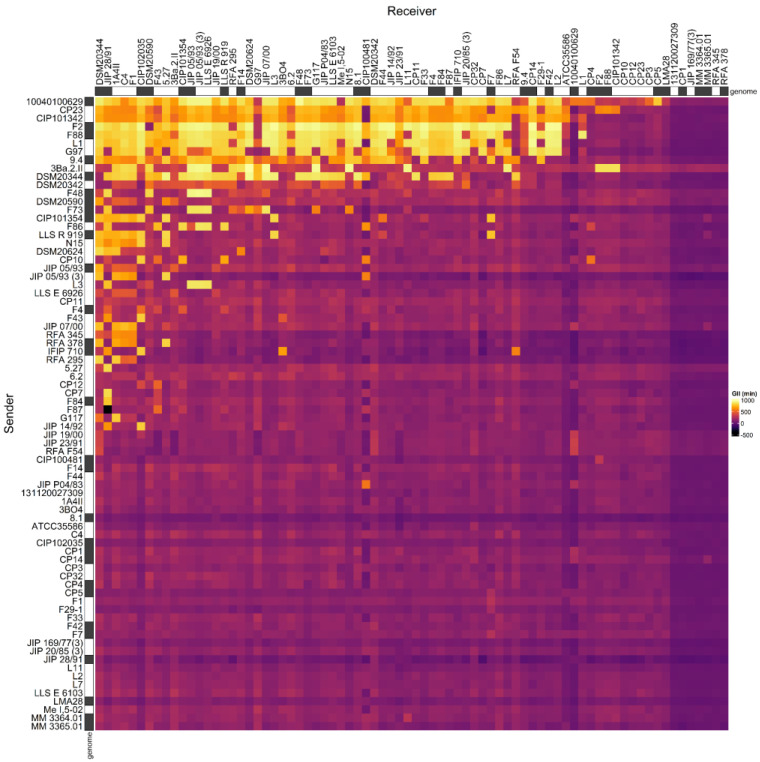
Adjacency matrix representing the GII inhibition values resulting from pairwise competition assays in *C. maltaromaticum*. The matrix was constructed with the data produced for 73 strains by [33] and the three additional strains *C. maltaromaticum* 10040100629, 3Ba.2.II, and DSM20590. The supernatant of sender strains (in rows) was added to cultures of the receiver strains (in columns) in order to measure their impact on the growth of the receivers. The color scale from purple to yellow represents increasing GII values. On the *X*-axis the strains are sorted in the descending order of sender degree from the left to the right. On the *Y*-axis, the strains are sorted in descending order of receiver degree, from the top to the bottom. The strains for which the genome was analyzed in this study are indicated with a grey box next to the name of the strains.

We previously described the antagonistic properties of a set of *C. maltaromaticum* strains by considering the data in a qualitative binary approach, without considering the intensity of inhibitions [33]. Here, we reconsidered these data by taking into account the inhibition intensities. To do so, the data produced by [33], as well as the data obtained for three additional strains (*C. maltaromaticum* 10040100629, 3Ba.2.II, and DSM20590) were used to construct an adjacency matrix describing the intensity of inhibition between each pair of strains that can be formed from the set of 76 selected *C. maltaromaticum* strains (Figure 1).

**Figure 2:**
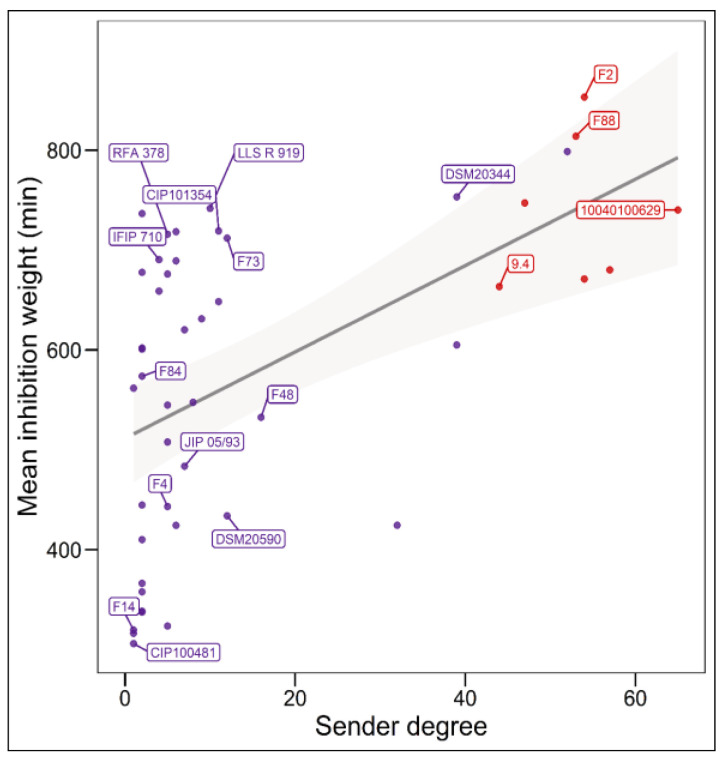
Relationship between the sender degree, the inhibitory potency, and the ability to inhibit *L. monocytogenes* EGDe. The sender degree refers to the number of receiver *C. maltaromaticum* strains inhibited by a given sender *C. maltaromaticum* strain. The mean inhibition weight is the mean of GII values corresponding to inhibitions and recorded for each sender strain. Only strains for which the sender degree is different from 0 and for which the genome was analysed in this study are labelled. Simple linear regression modelling was performed (*p*-value = 8.8 × 10^−5^, R^2^ = 0.29), the grey shaded area denotes the 95% confidence intervals (*p*-value < 0.05).

The analysis of the relationship between the mean intensity of inhibition of *C. maltaromaticum* receiver strains and the sender degree (the number of strains inhibited by a given strain, i.e., the width of the inhibitory spectrum) revealed that the intraspecific inhibition spectra are related to the inhibition potency. Indeed, the broader the spectrum of inhibition of *C. maltaromaticum* strains, the higher the potency of inhibition (*p*-value < 8.8 × 10^−5^) (Figure 2). In addition, the strains that were able to strongly inhibit *L. monocytogenes* EGDe, including the strains F2, F88, 10040100629, and 9.4, show a wide spectrum of inhibition of conspecific strains. However, the linear model only explains approximately 29% of the variability (R^2^ = 0.29). This is due to strains with low sender degrees and high inhibition potency, namely strains RFA 378, LLS R 919, IFIP 710, CIP 101354, and F73 (Figure 2). This indicates that strains with narrow intraspecific inhibitory spectra can also exhibit potent antimicrobial properties.

**Figure 3:**
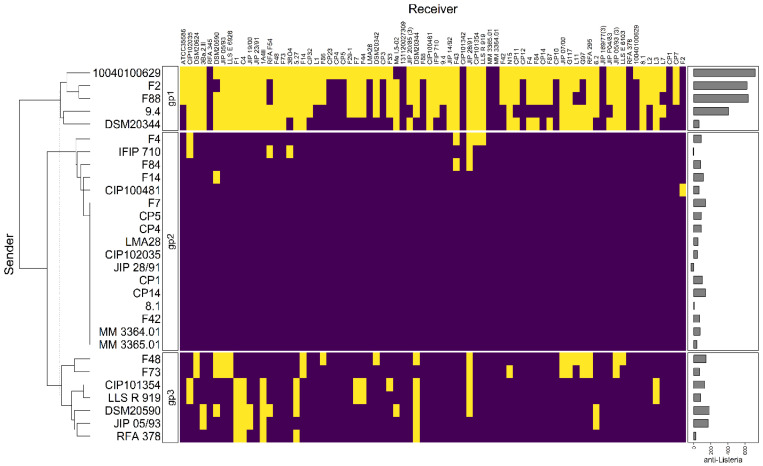
Hierarchical clustering analysis of the sender strains. The sender strains subjected to genome analysis in this study (in rows) were clustered based on their inhibition profiles of the *C. maltaromaticum* receiver strains (in columns). Yellow cells represent inhibition, purple cells non-inhibition. The clustering divides the senders into three groups, noted “gp1”, “gp2”, and “gp3”. The barplot annotation on the right represents the quantitative inhibition of *L monocytogenes* EGDe (in min) by the sender strains.

**Figure 4:**
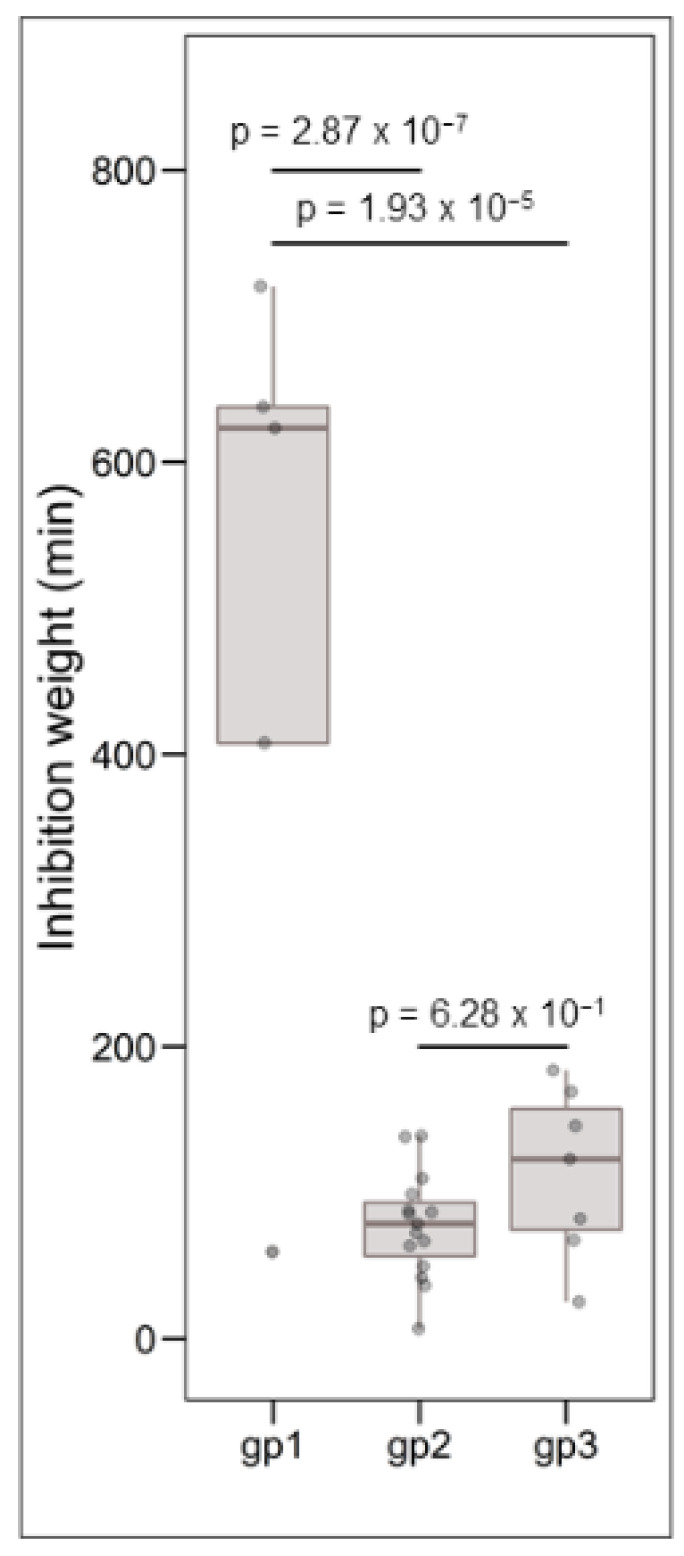
Boxplots representing the distribution of *L. monocytogenes* EGDe inhibition, the grouping variable being the group defined by hierarchical clustering (see Figure 3). The data were analysed by one-way ANOVA followed by a Tukey’s test. The resulting *p*-values are indicated above the compared groups.

Clustering analysis was performed on the basis of intraspecific inhibition spectra of the 29 strains that were subsequently selected for genome mining. The strains were clustered into three groups: gp1, gp2, and gp3 encompassing 5, 17, and 7 strains, respectively (Figure 3). The strains from gp2 and gp3 have a narrow inhibitory spectrum of *C. maltaromaticum* strains and weakly inhibit *L. monocytogenes* EGDe. Conversely, the strains that belong to gp1 have a wide spectrum of inhibition of *C. maltaromaticum* as well as a high potency of inhibition of *L. monocytogenes* EGDe (*p*-values < 0.001, Figure 4). However, not all strains of gp1 are characterized by high anti-*L. monocytogenes* EGDe potency since among the five strains, the strain DSM20344 is a weak inhibitor of the pathogen (Figure 3).

### 3.2. Bacteriocin Gene Clusters

**Figure 5:**
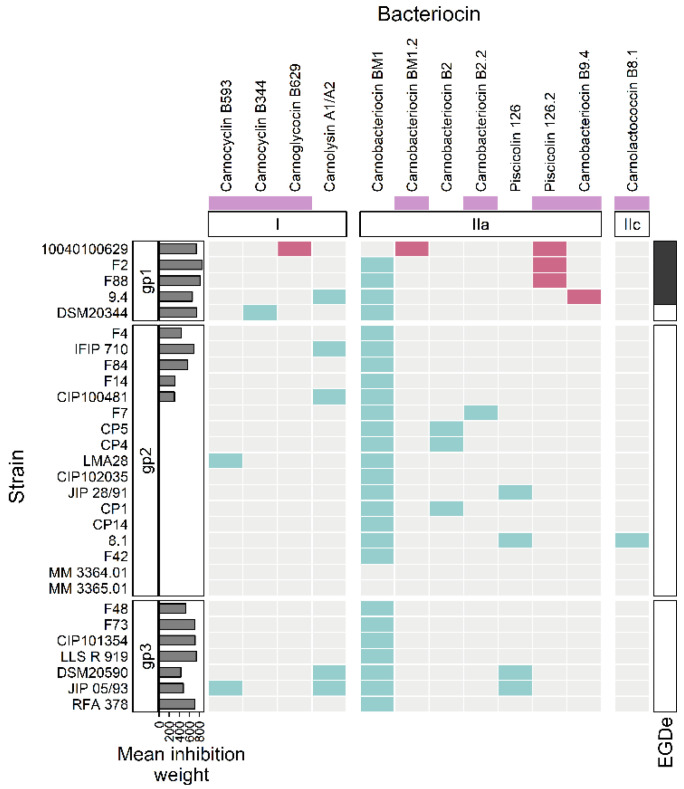
Heatmap representing the occurrence of bacteriocin BGCs in the genomes. The strains of *C. maltaromaticum* (in rows) are grouped according to the groups “gp1”, “gp2”, and “gp3”. The considered bacteriocin BGCs (in columns) are grouped according to the class of bacteriocin to which they belong (class I, IIa, IIc). The mauve boxes, below the bacteriocin names, denotes newly described bacteriocins in this study. The barplot annotation on the left represents the mean inhibition weight (in min) of *C. maltaromaticum* receiver strains by the sender strains. In the annotation block on the right, black boxes indicate inhibition of *L. monocytogenes* EGDe by the *C. maltaromaticum* strains. The presence of a bacteriocin BGC is indicated by turquoise or red cells. The red cells denote bacteriocin BGCs specifically found in *C. maltaromaticum* strains with anti-*L. monocytogenes* activity, the other BGC are denoted in turquoise.

A subset of 29 strains with varying sender and receiver profiles were selected (Figure 1) for genome mining in order to identify potential bacteriocin BGCs. Overall, 13 bacteriocin structural genes encoding 12 bacteriocins were predicted in the genome of these strains (Figure 5, Table 1, Tables S2 and S3).

More precisely and according to the classification proposed by Alvares-Siero et al. [4], five structural genes would encode four class I bacteriocins: two head-to-tail cyclic peptides (carnocyclin B593 and carnocyclin B344), one glycocin (carnoglycocin B629) and one two-component lantibiotic (carnolysin A1-A2, encoded by two Coding DNA Sequences, CDSs). Eight clusters were associated with class II bacteriocins, among which seven structural genes of class IIa (carnobacteriocin BM1 and BM1.2, carnobacteriocin B2 and B2.2, piscicolin 126, and 126.2, and carnobacteriocin B9.4), and one structural gene related to class IIc (carnolactococcin B8.1).

Among the 12 possible bacteriocin homologs found in this study, four have 100% identity with previously described bacteriocins: carnolysin A1-A2, carnobacteriocin BM1, carnobacteriocin B2, and piscicolin 126. The other eight bacteriocins have newly identified sequences either because they are variants with slight changes in the amino acid sequences compared to known bacteriocins (carnobacteriocin BM1.2, carnobacteriocin B2.2, and piscicoline 126.2), or because they are distantly related to other bacteriocins (carnocyclin B593, carnocyclin B344, carnoglycocin B629, carnobacteriocin B9.4, and carnolactococcin B8.1).

#### 3.2.1. Class I

**Figure 6:**
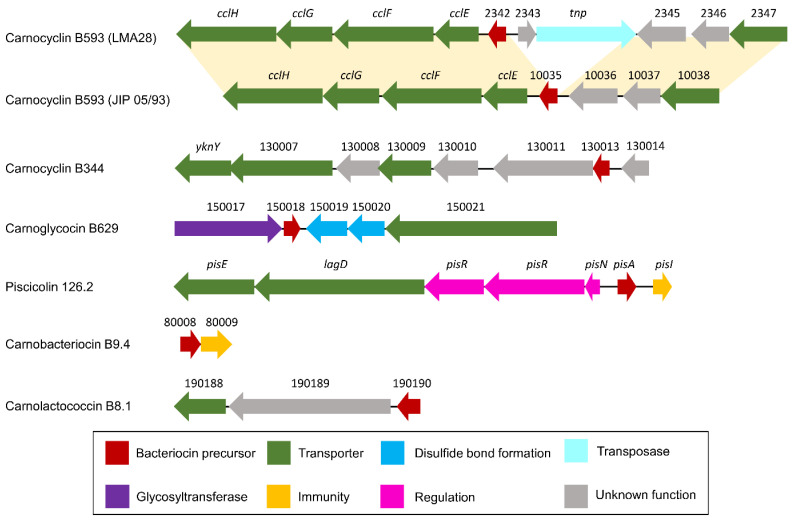
Schematic representation of bacteriocin BGCs. Below the arrows which represent CDSs, the short names of the locus tags are indicated (the prefix have been omitted: “BN424_”, “CMALT398_v1_”, “CMDSM20344_v1_”, “CMALT430_v1_”, “CMALT94_v1_”, “CMALT81_v1_” for LMA28, JIP 05/93, DSM20344, 10040100629, 9.4, and 8.1, respectively). The yellow shading connects homologous regions between BGCs.

The putative carnocyclin B593 (CMALT398_v1_10035, BN424_2342) and carnocyclin B344 (CMDSM20344_v1_130013) are both annotated as belonging to the uberolysin/carnocyclin family of circular bacteriocins (Table 2). Carnocyclin B593 has been predicted in the genome of *C. maltaromaticum* LMA28 and JIP 05/93, while carnocyclin B344 has been predicted in the genome of the strain DSM20344 (Figure 5). Circular bacteriocins have N-to-C- terminal covalent linkages forming a circular peptide backbone [54]. As expected for group “i” of circular bacteriocins (please refer to [54] for group definition), the predicted isoelectric point is high (8.9 and 10.1 for carnocyclin B593 and B344, respectively). As for other circular bacteriocin BGCs, most of the CDSs encode proteins with membrane-associated function (seven out of nine or eleven CDSs for carnocyclin B593, depending on the strain, and six out of nine CDSs carnocyclin B344), of which several would be involved in transport across the membrane (Figure 6, Table 2).

Genes homologous to *cclF* and *cclE* of the carnoclycin A BGC were found in the carnocyclin B593 BGC and would encode transporters [55] (Figure 6, Table 2). No CDS clearly associated with an immunity function has been identified. In addition, and surprisingly, no homolog of the DUF95 family was found, neither in the vicinity of the bacteriocin structural genes nor elsewhere in the genome of these two strains. Yet, the presence of such a gene is conserved in clusters of described circular bacteriocins. In addition, this protein is suspected to be directly involved in the cyclization of these circular bacteriocins even if experimental data are missing in the literature [54]. This suggests that these bacteriocins could be linear instead of circular.

**Figure 7:**
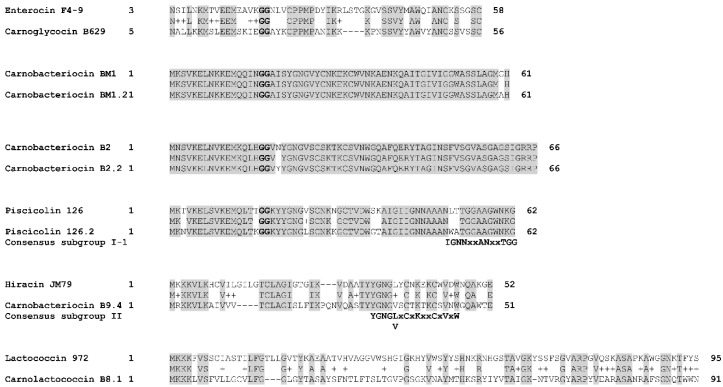
Alignment of bacteriocins homologs. The alignment was performed with BLASTp by using the BLOSUM62 matrix. The double glycines of the leader peptides are indicated in bold. The consensus sequences described for class IIa bacteriocins by [57] are indicated below the alignments. Identical amino acids are shaded in grey. Chemically similar amino acids are connected by a “+” symbol.

The putative carnoglycocin B629 (CMALT430_v1_150018) from strain *C. maltaromaticum* 10040100629 is homologous to the glycocin Enterocin F4-9 (query coverage 83%, identity 52%, similarity 64%, Figure 7). Glycocins are glycosylated peptides where the carbohydrate group is covalently linked to a serine/threonine residue (O-linked) or a cysteine residue (S-linked). In addition, glycocins have four cysteine residues that are engaged in two disulfide bonds, thus stabilizing the helix-loop-helix structures of the polypeptide [56]. The sequence of carnoglycocin B629 contains four Cys residues (Cys26, Cys50, Cys56, and Cys64, Table S2) that could be involved in two disulfide bonds. Consistently, two CDSs potentially encoding thiol-disulfide isomerases (CMALT430_v1_150019 and CMALT430_v1_150020, Figure 6, Table 2) were predicted in the BGC. These enzymes could be involved in the formation of two disulfide bonds in carnoglycocin B629. Upstream of the carnoglycocin B629 CDS, a CDS encoding a glycosyltransferase (CMALT430_v1_150017) was predicted and could be involved in the glycosylation of the bacteriocin (Figure 6, Table 2). Twelve possible sites for O-glycosylation are present in the sequence of carnoglycocin B629 (Ser12, Ser17, Ser39, Ser40, Ser51, Ser52, Ser54, Ser55, Ser57, Thr59, Thr60, and Thr63, Table S2). The carnoglycocin B629 BGC also encompasses a CDS (CMALT430_v1_10021) encoding a peptidase domain-containing ABC transporter that may be involved in the export and signal peptide removal of the bacteriocin (Figure 6, Table 2).

The carnolysin A1-A2 BGC was identified in the genome of five strains, namely *C. maltaromaticum* 9.4, IFIP 710, CIP100481, DSM20590, and JIP 05/93 (Figure 5). The primary structure of the bacteriocins shares 100% identity with the carnolysin A1-A2 described in the databases (query coverage of 100%). Carnolysins A1 and A2 combine to form a two-peptide lantibiotic synthesized by *C. maltaromaticum* C2. It is an extensively modified bacteriocin containing D-alanine and D-aminobutyrate derived from serine and threonine, respectively. This bacteriocin shows antimicrobial activity against other LAB, such as *Lactilactobacillus sakei*, as well as *Listeria innocua* and *L. monocytogenes* [58].

#### 3.2.2. Class IIa

Seven class IIa bacteriocins were predicted: carnobacteriocin BM1 and BM1.2, carnobacteriocin B2 and B2.2, piscicolin 126 and 126.2, and carnobacteriocin B9.4 (Figure 5). Class IIa bacteriocins are heat stable, non-modified, cationic, hydrophilic, and hydrophobic peptides that are exported thanks to a double–glycine-containing leader peptide. They classically have a molecular weight of less than 10 kDa [59,60]. The pediocin-like bacteriocins are particularly active against *Listeria* spp. The N-terminal region contains two or four cysteine residues joined by one or two disulfide bridges, and a conserved YGNGV-C motif (less frequently the valine is replaced by leucine) while the C-terminus is less conserved [57].

The variant carnobacteriocin BM1.2 was predicted in the genome of the strain *C. maltaromaticum* 10040100629 and exhibits an alanine residue instead of glycine at position 60 (Figure 7). Carnobacteriocin B2.2 was found in the genome of *C. maltaromaticum* F7 (Figure 5) and presents a tyrosine residue instead of asparagine at position 20 (Figure 7). The variant piscicolin 126.2 was found in the genome of the three strains *C. maltaromaticum* 10040100629, F2, and F88. It shows eight substitutions of which two are localized in the leader peptide (T3N, T16K), and could influence the export efficiency. The remaining six other substitutions are localized in the mature bacteriocin, including a V25L substitution in the YGNGV motif. Although piscicolin 126.2 exhibits five substitutions compared to piscicolin 126, it contains the consensus sequence IGNNxxANxxTGG which is typically found in the subgroup I-1 of class IIa bacteriocins [57]. It can be noticed that the nucleotide sequence of the piscicolin 126.2 BGC, and more specifically of the intergenic region between *pisN* and *pisA* (Figure 6), exhibit 94% and 88% identity, respectively, with the piscicolin 126 BGC.

The genome of the strain *C. maltaromaticum* 9.4 is predicted to encode the class IIa carnobacteriocin B9.4 (CMALT94_v1_80008, Figure 5, Table 1) which is a homolog of hiracin JM79 (query coverage 70%, 56% identity, Figure 7). Hiracin JM79 is a bacteriocin with potent anti-*L. monocytogenes* activity [61]. The CDS CMALT94_v1_80008 is localized next to a homolog of the leucocin A immunity encoding gene (CMALT94_v1_80009, Figure 6). The sequence of carnobacteriocin B9.4 matches with the consensus sequence YGNGL(V)xCxKxxCxVxW described for the group II of class IIa bacteriocins to which hiracin JM79 belongs [57]. Similar to hiracin JM79 and other bacteriocins of group II, carnobacteriocin B9.4 is predicted to contain a *sec* signal peptide of 29 amino acid residues suggesting that this bacteriocin is secreted thanks to the *sec* translocase [57]. The two CDSs encoding carnobacteriocin B9.4 and the immunity factor belong to a genomic island of 27 kbp which encompasses 40 CDSs and is localized downstream of a Leu-tRNA gene. This genomic island contains CDSs potentially encoding an integrase, an excisionase, a conjugation protein (FtsK-like DNA-translocation protein), and other functions typically found in mobile genetic elements (CDSs encoding a LPXTG-surface protein and site-specific DNA-methyltransferase), suggesting that the CDS encoding carnobacteriocin B9.4 is localized within an Integrative and Conjugative Element (ICE) and can be subjected to horizontal transfer [62].

#### 3.2.3. Class IIc

The CDS CMALT81_v1_190190, from the genome of *C. maltaromaticum* 8.1, is predicted to encode a class IIc bacteriocin, called carnolactococcin B8.1 (Table 1). Class IIc bacteriocins are small, heat-stable, leaderless peptides that do not undergo any post-translational modifications [54]. Carnolactococcin B8.1 shows a weak resemblance with lactococcin 972 (query coverage 100%, identity 36%, similarity 56%, Figure 7). The BGC predicted in the genome of *C. maltaromaticum* 8.1 comprises one putative ABC-type transporter that could be involved in bacteriocin export and a membrane protein of unknown function (Figure 6, Table 2).

### 3.3. NRPS-BGC

**Figure 8:**
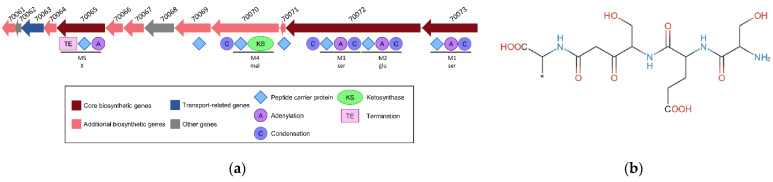
NRPS-PKS BGC was predicted in the genome of the strain *C. maltaromaticum* 10040100629. (**a**) Representation of the genetic organization where the CDSs are represented by arrows. The domains predicted in the encoded proteins are represented below the CDSs. The locus tag is indicated above the CDSs, the prefix “CMALT430_v1_” of the locus tag was omitted; (**b**) core structure of the predicted metabolite.

An NRPS-PKS BGC of 26.3 kbp comprising 13 CDSs was predicted in the genome of *C. maltaromaticum* 10040100629 (Figure 8, Table 2). Altogether, the CDSs encode seven peptide carrier protein domains, four adenylation domains, five condensation domains, one ketosynthase domain, and one termination domain (Figure 8a). Five modules (Figure 8a) were predicted to synthesize a metabolite with the following structure: (ser) + (glu − ser) + (mal) + (X) (Figure 8b). Additional CDSs whose function is compatible with the synthesis of specialized metabolites are also present and encode one 4’-phosphopantetheinyl transferase, two thioesterases, two Acyl-CoA dehydrogenases, and one aminopeptidase (Table 2).

### 3.4. Genome Clustering

**Figure 9:**
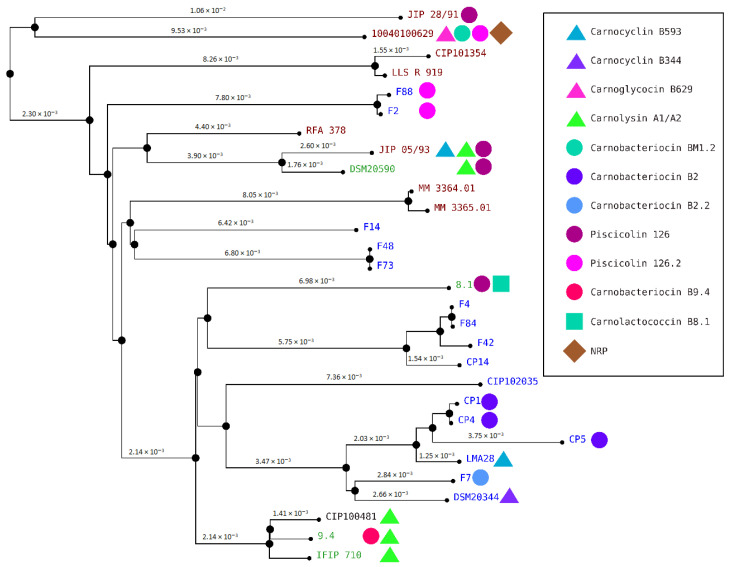
*C. maltaromaticum* genome clustering. The names of the *C. maltaromaticum* strains are indicated, in blue, green, dark red, and black when the strains were isolated from a dairy product, other food products, diseased fish, and human blood, respectively. Next to strain names, the symbols indicate the bacteriocin and the NRP predicted from the genome of the strains. The bacteriocin BM1 is not represented.

Genome clustering revealed that the carnobacteriocin B2 BGC is distributed among the closely related strains CP1, CP4, and CP5 (Figure 9). By contrast, the carnocyclin B593, the carnolysin A1/A2, and the piscicolin 126 BGCs are distributed in several clades. Indeed, the carnocyclin B593 BGC is present in two distant strains: JIP 05/93 and LM128 (Figure 9). Similarly, the carnolysin A1/A2 BGC is present in two clades: one formed by the strains JIP 05/93, and DSM20590, and the other by CIP100481, 9.4, and IFIP 710; and the piscicolin 126.2 BGC in other two clades: one formed by the strain 10040100629 and the other encompassing the strains F2 and F88. Furthermore, the piscicolin 126 BGC is present in three lineages: one formed by JIP 28/91, the second by JIP 05/93 and DSM20590, and the third by 8.1 (Figure 9). The distribution of these BGCs in multiple lineages suggests that they evolved by horizontal transfer.

### 3.5. Comparisons of Antimicrobial Phenotypes and Genome Predictions

The three strains *C. maltaromaticum* 10040100629, F2, and F88, which belong to gp1, are characterized by a potent anti-*L. monocytogenes* EGDe activity and a high sender degree (Figure 2 and Figure 3). As these three strains are the only ones to potentially produce piscicolin 126.2 (Figure 5), it is likely that their remarkable antimicrobial properties, and in particular their anti-*L. monocytogenes* activity is at least in part due to the production of piscicolin 126.2.

The strain *C. maltaromaticum* 10040100629 is characterized by a higher inhibition potency of *L. monocytogenes* EGDe compared to strains F2 and F88 (Figure 2). Interestingly, this strain would produce two bacteriocins in addition to piscicolin 126.2: carnoglycocin B629, and carnobacteriocin BM1.2, as well as an NRP-PK compound. All or part of these compounds could be responsible for the higher potency of the strain 10040100629.

According to the hierarchical clustering analysis, the inhibition profile of strain 9.4 is relatively distant from the other three 10040100629, F2, and F88, even though all these strains belong to gp1 and exhibit anti-*L. monocytogenes* activity (Figure 3). Interestingly, strain 9.4 is the only one that potentially produces carnobacteriocin B9.4 which could therefore be responsible for the potent anti-*L. monocytogenes* activity of this strain.

Several strains are predicted to produce the single bacteriocin BM1. However, it is described in the literature that the production of this bacteriocin is highly dependent on the presence of carnobacteriocin B2 BGC, which contains the CDSs required for the synthesis of the two-component regulatory system CbnRK which induces the expression of both carnobacteriocin B2 and BM1 [63]. It is therefore unlikely that the anti-*C. maltaromaticum* activity of the strains for which the carnobacteriocin BM1 is the only bacteriocin-encoding CDS found in the genome, namely the strains F4, F84, F14, F48, F73, CIP101354, and LLS R 919 (Figure 5), results from the production of this bacteriocin. In addition, all the strains carrying the CDS encoding carnobacteriocin BM1 (26 strains out of 29) also have the associated CDS encoding the immunity protein, thus reinforcing this hypothesis. Furthermore, the strains CIP102035, CP14, and F42 have no detected antimicrobial activity under our experimental conditions even though they possess the CDS encoding carnobacteriocin BM1. All these observations suggest that the anti-*C. maltaromaticum* activity of the strains F4, F84, F14, F48, F73, CIP101354, F73, CIP101354, and LLS R 919 is not due to BM1 but rather to one or several compounds produced thanks to BGCs that were not detected by genome mining.

The strains F7, CP5, CP4, LMA28, JIP28/91, CP1, and 8.1 have a null sender degree (Figure 3), even though they were predicted to produce other bacteriocins than BM1, namely carnocyclin B593, carnobacteriocin B2 and B2.2, piscicolin 126, and carnolactococcin B8.1 (Figure 5).

## 4. Discussion

In this study, the antimicrobial properties of *C. maltaromaticum* were investigated by combining interference competition network analysis and genome mining. From the inhibition data obtained with 76 *C. maltaromaticum* strains, the relationship between the potency of inhibition, the sender degree (i.e., inhibitory spectrum), and the anti-*L. monocytogenes* EGDe properties were studied. The genome of a selection of 29 strains, representative of the diversity of the inhibition profiles, was analyzed in order to predict the genes with functions associated with antimicrobial activity.

The results have shown that the potency of inhibition tended to increase when the inhibition spectrum broadened. In addition, the strains that were able to effectively inhibit *L. monocytogenes* EGDe also exhibited a broad inhibition spectrum of *C. maltaromaticum* strains. This suggests that the use of *C. maltaromaticum* strains with anti-*L. monocytogenes* for biopreservation purposes may reduce the diversity of autochthonous *C. maltaromaticum* populations in food microbiota. This would be in agreement with a previous report showing that bioprotective *Lactilactobacillus curvatus* markedly decrease the diversity of the metabolically active microbial community in fermented sausages [64].

Although the potency of inhibition tended to increase when the sender degree increased, several strains were characterized by high potency of inhibition and narrow anti-*C. maltaromaticum* spectrum. This suggests that the antimicrobial compounds involved in these inhibitions exhibit high molecular specificity and target receptors characterized by inter-strain variability. Little is known about the bacteriocins involved in conspecific inhibitions in LAB since the majority of studies are conducted with the perspective of engineering biopreservation systems targeting unwanted microorganisms. However, it is known that bacteriocins produced by LAB can specifically target bacteria that are closely related to the producers. For instance, the bacteriocin mesenterocin 52B is produced by *Leuconostoc mesenteroides* subsp. *mesenteroides* FR52 only inhibits bacteria from the genus *Leuconostoc* [65].

The mining of 29 genomes of *C. maltaromaticum* allowed us to predict 12 bacteriocins and one NRPS-PKS BGCs. This is consistent with previous genome mining analysis in the species *C. maltaromaticum* and *C. divergens* [66]. This suggests that bacteriocins are the main antimicrobial specialized metabolites produced by *C. maltaromaticum*. Similarly, LAB are mainly reported to produce specialized metabolites of the type bacteriocin [67]. By contrast, few BGCs potentially encoding NRPS/PKS are described in the literature for LAB [5,6,7]. The tremendous amount of available bacterial genomic and metagenomic data provided opportunities to investigate the distribution of BGCs in bacterial taxa. Genome mining of the human microbiome project’s reference genome database revealed that bacteriocin BGCs are mainly found in the phyla *Firmicutes* and *Proteobacteria* and less frequently encountered in other phyla including *Actinobacteria* [68]. Conversely, NRPS/PKS BGCs are widely encountered in *Actinobacteria*, *Proteobacteria*, *Verrumicrobia,* and *Bacteroidetes* [69]. In the phylum *Firmicutes*, PKS BGC is preferably encountered in the order *Bacillales* of the phylum *Firmicutes* [70]. The reason why these genes are heterogeneously distributed is not known.

The genome of *C. maltaromaticum* 10040100629 was predicted to contain an NRPS-PKS BGC allowing the potential biosynthesis of a compound with the structure (ser) + (glu − ser) + (mal) + (X). The genome of *Carnobacterium divergens* V41 also contains a genomic island of 39 kbp encompassing three core synthetic CDSs which encodes seven modules that would synthesize a compound with the following structure: (mal) + (leu − D-ser) + (pro − X − cys − gly) [5]. Nonribosomal compounds vary from about 3 to 15 amino acids in length and can exhibit a linear, cyclic, or branched-cyclic structure [71,72]. It has been previously described that NRPs as short as tetrapeptides can exhibit antimicrobial activity [73], opening the possibility that the specialized metabolites potentially produced by *C. maltaromaticum* 10040100629 and *C. divergens* V41 have antimicrobial activity. However, the biological properties of NRP/PK compounds are not exclusively restricted to antimicrobial activities. In *L. lactis* KF147, an NRPS-PKS system improves tolerance to reactive oxygen species [6]. Further studies are required to better understand the role of these compounds in LAB.

Among the bacteriocins predicted in this study, piscicolin B126.2 is likely involved in the remarkable antimicrobial activity of the strains *C. maltaromaticum* 10040100629, F2, and F88. The sequence of piscicolin 126.2 precursor shows eight amino acid substitutions. It has been shown that amino acid substitutions can change the stability, potency, and spectrum of activity of class IIa bacteriocins [74]. Amino acid changes can either reduce or increase the potency of the bacteriocins. For instance, the addition of lysine at the C-terminus and T20K mutants of sakacin P shows increased cell binding and potency [74,75]. The increase in bacteriocin activity thanks to amino acid substitutions was also observed for nisin [76]. Alternatively, the high activity of the strains *C. maltaromaticum* 10040100629, F2, and F88 could result from high expression of piscicolin 126.2 since multiple point mutations are present in the piscicolin 126.2 BGC compared to the already described piscicolin 126 BGC, including in the intergenic region which likely contains the promoters the bacteriocin structural gene (*pisA*) and the export, regulation, and maturation system (*pisNKR, lagD*, and *pisE*, Figure 6).

Several strains were predicted to produce known bacteriocins, yet these strains have no antimicrobial activity under our experimental conditions. It has been described that the regulation of piscicolin 126 BGC is variable, depending on the strains considered. More precisely, the strain *C. maltaromaticum* JG126, produces piscicolin 126 regardless of the cultivation temperature, while the strain *C. maltaromaticum* UAL26 produces the bacteriocin only at temperatures below 19 °C. When this latter strain is cultivated at 25 °C, it does not produce piscicolin 126 [77]. Apart from the strains containing the piscicolin 126 BGC, other strains such as *C. maltaromaticum* CP5 carry the carnobacteriocin B2 BGC in their genome and yet were not found as inhibitory. The strain *C. maltaromaticum* CP5 was studied in our laboratory 30 years ago and revealed potent anti-*L. monocytogenes* activity [78]. However, our latest attempts to show any antimicrobial activity with this strain were not successful. The historical frozen culture stocks of this strain are no longer available in our laboratory. The instability of bacteriocin production was also observed for *Lactobacillus sake* L45, for which the propagation in a liquid medium results in the emergence of non-bacteriocin-producing variants at high frequency [79].

Several strains were found to exhibit potent anti-*C. maltaromaticum* with a narrow spectrum. This activity could be attributable to the production of several bacteriocins (carnobacteriocin B593, carnolysin A1/A2, and piscicolin 126), alone or in combination for the strains IFIP 710, and CIP 100481, DSM20590, and JIP 05/93 (Figure 5). However, no BGC candidates were predicted for the other anti-*C. maltaromaticum* strains F4, F84, F14, F48, F73, CIP101354, LLS R 919, and RFA 378. It can be noticed that among these strains, RFA 378, LLS R 919, CIP101354, IFIP 710, and F73 have a potent anti-*C. maltaromaticum* activity (Figure 2). This suggests that genome mining would not be able to identify BGCs allowing the production of antimicrobial substances acting on conspecific strains. The basis of functional prediction in genome mining is the availability of experimental data providing information on the function of genes. In the field of antimicrobials produced by LAB, the literature describing data obtained from wet experiments is tremendously dedicated to the characterization of compounds inhibiting unwanted microorganisms, such as *L. monocytogenes*. This documentary bias may have led to missing genes encoding the synthesis of antimicrobial substances whose sequence properties are unrelated to those already described in the databases. However, these substances could play an important role in ecosystems by contributing to the structuring of microbial food communities. Recent advances in the identification techniques, in particular those involving artificial intelligence such as deep learning approaches [80], could be a real game changer in the area.

## 5. Conclusions

*C. maltaromaticum* is a species of high interest for dairy products, especially for biopreservation purposes, that has been extensively studied for the production of bacteriocins. However, it has been shown here using a combined approach of inhibition network analysis and genomics that this species harbors an unexplored potential that could be of direct interest specifically for biopreservation but also more globally for the understanding and engineering of dairy ecosystems. The further use of these high throughput approaches should lead to new technological innovations for better control of the quality of dairy products.

## Figures and Tables

**Table 1:** Bacteriocins predicted from the genome of 29 strains of *C. maltaromaticum*.

Class	Family	Name		Strain
I Cyclic peptide	circularin A/uberolysin	Carnocyclin	B593	JIP 05/93
			B344	DSM20344
I Glycocin		Carnoglycocin	B629	10040100629
I Lantibiotic	Two component bacteriocin	Carnolysin	A1	JIP 05/93; DSM20590; CIP100481; 9.4; and IFIP 710
			A2	
IIa	pediocin-like	Carnobacteriocin	BM1	All excluding MM 3364.01; MM 3365.01; and 10040100629
			BM1.2	10040100629
		Piscicolin	126	DSM20590; JIP 28/91; JIP 05/93; and 8.1
			126.2	10040100629
		Carnobacteriocin	B2	CP1; CP4; and CP5
			B2.2	F7
		Carnobacteriocin	B9.4	9.4
IIc	Lactococcin 972	Carnolactococcin	B8.1	8.1

**Table 2:** BGCs potentially encoding the synthesis of specialized metabolites.

Metabolite	Locus Tag	Gene	Putative Function
Carnocyclin B593	CMALT398_v1_10031/BN424_2338	*cllH*	ABC transporter, permease protein
	CMALT398_v1_10032/BN424_2339	*cclG*	Uncharacterized ABC transporter ATP-binding protein YknY
	CMALT398_v1_10033/BN424_2340	*cclF*	Membrane-fusion protein/RND family efflux transporter
	CMALT398_v1_10034/BN424_2341	*cclE*	Putative membrane protein
	**CMALT398_v1_10035/BN424_2342**		**Bacteriocin precursor, circularin A/uberolysin family protein**
	BN424_2343		Hypothetical protein
	BN424_2344	*tnp*	Transposase
	CMALT398_v1_10036		Membrane protein of unknown function
	CMALT398_v1_10037		Conserved membrane protein of unknown function
	CMALT398_v1_10038		ABC-type multidrug transport system, ATPase component/(ABC) transporter
	CMDSM20344_v1_130006	*cclG*	Uncharacterized ABC transporter ATP-binding protein YknY
Carnocyclin B344	CMDSM20344_v1_130007		Periplasmic component of efflux system
	CMDSM20344_v1_130008		Conserved membrane protein of unknown function
	CMDSM20344_v1_130009		ABC transporter domain-containing protein
	CMDSM20344_v1_130010		Conserved membrane protein of unknown function
	CMDSM20344_v1_130011		Conserved membrane protein of unknown function
	CMDSM20344_v1_130012		Protein of unknown function
	**CMDSM20344_v1_130013**		**Bacteriocin precursor, circularin A/uberolysin family protein**
	CMDSM20344_v1_130014		Protein of unknown function
Carnobacteriocin B9.4	**CMALT94_v1_80008**		**Bacteriocin precursor**
	CMALT94_v1_80009		putative immunity protein
Carnoglycocin B629	CMALT430_v1_150017		Glycosyltransferases involved in cell wall biogenesis/Glycosyltransferase, family 2
	**CMALT430_v1_150018**		**Bacteriocin precursor**
	CMALT430_v1_150019		Thiol-disulfide isomerase and thioredoxins, lipoprotein signal peptide
	CMALT430_v1_150020		Thiol-disulfide isomerase and thioredoxins
	CMALT430_v1_150021		Peptidase domain-containing ABC transporter
Carnolactococcin B8.1	CMALT81_v1_190188		Bacteriocin ABC transporter ATP-binding protein
	CMALT81_v1_190189		Membrane protein of unknown function
	**CMALT81_v1_190190**		**Bacteriocin precursor, lipoprotein signal peptide**
NRP-PK compound (ser) + (glu-ser) + (mal) + (X)	CMALT430_v1_70061		SMCOG1012:4’-phosphopantetheinyl transferase, ACPS
	CMALT430_v1_70062		Protein of unknown function
	CMALT430_v1_70063		Conserved membrane protein of unknown function, transport
	CMALT430_v1_70064		putative Surfactin synthase thioesterase subunit, SMCOG1004: thioesterase
	CMALT430_v1_70065		SMCOG1002: AMP-dependent synthetase and ligase, AMP-binding, PCP, Thioesterase
	CMALT430_v1_70066		Putative Acyl-CoA dehydrogenase, Acyl-CoA_dh_1
	CMALT430_v1_70067		Putative Acyl-CoA dehydrogenase, Acyl-CoA_dh_N; Acyl-CoA_dh_M; Acyl-CoA_dh_1; SMCOG1006:acyl-CoA dehydrogenase
	CMALT430_v1_70068		Putative predicted aminopeptidases
	CMALT430_v1_70069		PP-binding, PCP
	CMALT430_v1_70070		Putative carrier domain-containing protein, SMCOG1022:Beta-ketoacyl synthase, PKS_KS, PCP, Condensation_LCL
	CMALT430_v1_70071		PP-binding, PCP
	CMALT430_v1_70072		Condensation_LCL, AMP-binding, PCP, Condensation_LCL, AMP-binding, PCP, Condensation_LCL
	CMALT430_v1_70073		Condensation_LCL, AMP-binding, PCP

## Data Availability

The data and the code used in R are available on GitHub: https://github.com/fredericborges/network_genome_antimic (accessed on 21 July 2022).

## Supplementary Materials

The following supporting information can be downloaded at: https://www.mdpi.com/article/10.3390/microorganisms10091794/s1, Table S1: Carnobacterium maltaromaticum strains used in this study; Table S2: Sequences of the bacteriocins predicted from the genome of 29 strains of *C. maltaromaticum*; Table S3: CDSs encoding structural bacteriocins in *C. maltaromaticum* strains. References in Supplementary Materials can be found in [18,33,81,82,83,84,85,86,87,88,89,90,91].
